# Rapid Plasma Reagin Tests of Serum, Cerebrospinal Fluid, and Aqueous Humor of Patients With Ocular Syphilis and AIDS

**DOI:** 10.1155/joph/5291594

**Published:** 2026-04-22

**Authors:** Yingjie Wang, Zhuyun Qian, Yong Tao, Wenjun Kong

**Affiliations:** ^1^ Department of Ophthalmology, Beijing Chaoyang Hospital, Capital Medical University, Beijing, 100020, China, ccmu.edu.cn; ^2^ Key Laboratory Jointly Built by the National Institute for Viral Disease Control and Prevention of China Center for Disease Control and Prevention, Beijing GIANTMED Medical Diagnostics Laboratory, Beijing, 101300, China; ^3^ National Engineering Research Center for Ophthalmology, Beijing, China; ^4^ Engineering Research Center of Ophthalmic Equipment and Materials, Ministry of Education, Beijing, China, meb.gov.tr; ^5^ Chinese Institutes for Medical Research, Beijing, 100069, China; ^6^ Department of Ophthalmology, Beijing You’an Hospital, Capital Medical University, Beijing, 100069, China, ccmu.edu.cn

**Keywords:** aqueous humor, ocular syphilis, rapid plasma reagin

## Abstract

**Purpose:**

The diagnosis of ocular syphilis in patients with acquired immunodeficiency syndrome (AIDS) remains challenging. This study aimed to evaluate the potential utility of aqueous humor (AH) RPR testing as an adjunctive diagnostic tool for assessing clinical manifestations and visual function in AIDS‐associated ocular syphilis.

**Methods:**

We conducted a retrospective observational cohort study of 31 patients (42 eyes) with ocular syphilis and AIDS treated at Beijing You’an Hospital between 2022 and 2025, based on comprehensive ophthalmic and systemic evaluations.

**Results:**

In patients with concurrent syphilis and AIDS, a higher pretreatment AH RPR reactivity level was associated with higher vitreous haze grades (OR = 4.10, 95% CI: 1.62–10.38, *p* = 0.003). Pretreatment serum, CSF, and AH RPR titers individually predicted macular chorioretinal involvement (AUC 0.76–0.85) and optic nerve involvement (AUC 0.62–0.78). Combining all three markers improved prediction for macular chorioretinal involvement (AUC 0.88) and for optic nerve involvement (AUC 0.79). RPR titers in serum, CSF, and AH were all significantly correlated with both pre‐ and posttreatment best‐corrected visual acuity, indicating that higher titers were associated with worse visual function.

**Conclusion:**

AH RPR can serve as a valuable adjunct for diagnosing ocular syphilis, as it indicates intraocular disease activity and informs treatment recommendations for the condition.

**Trial Registration:** Chinese Clinical Trial Registry: 2200056954

## 1. Introduction

Syphilis, caused by the spirochete *Treponema pallidum*, has increased markedly over the past decade, representing a persistent global public health threat [1]. The infection can involve multiple organ systems, including the eye [2]. Ocular syphilis, known as a great masquerader, may develop at any stage, affecting any ocular structure, and can lead to irreversible vision loss if not promptly recognized and treated [3]. Syphilis frequently coexists with acquired immunodeficiency syndrome (AIDS), the advanced stage of human immunodeficiency virus (HIV) infection, due to shared routes of transmission [4]. HIV‐related immunosuppression further alters the clinical course of syphilis, predisposing patients to atypical and more severe manifestations that complicate diagnosis and management and limit the availability of reliable prognostic indicators [5–7].

Currently, one of the diagnostic criteria for ocular syphilis relies on positive serum testing including treponemal tests such as *Treponema pallidum* particle agglutination (TPPA) and nontreponemal tests such as rapid plasma reagin (RPR) [8, 9]. However, in patients with HIV infection, immune suppression blunts the serologic response to *Treponema pallidum*, and conventional serologic tests often fail to reflect true disease activity. Cerebrospinal fluid (CSF) analysis is commonly used to complement serology by assessing neurological involvement, but studies have shown that its sensitivity in ocular syphilis is limited [10, 11].

Aqueous humor (AH) analysis has gained increasing attention as a diagnostic tool, owing to its direct sampling of the intraocular microenvironment, which allows for a more precise and targeted assessment of ocular pathologies [9, 12, 13]. However, the clinical significance of AH RPR testing in ocular syphilis remains to be determined. This study was designed to investigate the clinical utility of combined RPR testing in serum, CSF, and AH in patients with ocular syphilis and AIDS, with the aim of facilitating more accurate and timely diagnosis, thereby improving both visual function and overall patient outcomes.

## 2. Methods

### 2.1. Study Design and Population

This study was a retrospective study conducted on 31 patients (42 eyes) with AIDS diagnosed with ocular syphilis at the Department of Ophthalmology and the Center for HIV Infection of Beijing You’an Hospital. This study followed the Declaration of Helsinki and was approved by Beijing You’an Hospital. Patients who gave informed consent for participation were enrolled from January 2022 to March 2025.

All patients were diagnosed with AIDS based on established clinical and immunological criteria. HIV infection was confirmed by enzyme‐linked immunosorbent assay and chemiluminescence immunoassay, with positive results verified by Western blot. CD4^+^ T lymphocyte count and HIV viral load were measured, and AIDS was defined in patients with CD4^+^ T cell counts below 200 cells/μL. The diagnosis of ocular syphilis was established through comprehensive ophthalmological and laboratory evaluations. Ophthalmic assessments included best‐corrected visual acuity (BCVA), intraocular pressure, anterior segment examination, fundus photography, fundus autofluorescence, optical coherence tomography, fluorescein fundus angiography, and indocyanine green angiography. Laboratory evaluations included both treponemal and nontreponemal tests, with positive screening results confirmed by TPPA and disease activity monitored by RPR testing. Patients were screened to exclude other infectious diseases, such as tuberculosis and cryptococcosis. All participants had no prior ophthalmic surgery or laser treatment, and their visual function was normal before presentation.

### 2.2. Data Collection

Data on baseline demographic characteristics and medical history were obtained. Ophthalmological assessments included BCVA, intraocular pressure measurement, anterior segment examination with grading of anterior chamber and vitreous inflammation, and fundus examination.

To ensure diagnostic consistency, ocular endpoints were defined by clinical findings corroborated by multimodal imaging. First, macular chorioretinal involvement encompassed active inflammatory lesions such as (i) acute syphilitic posterior placoid chorioretinitis (ASPPC; yellowish‐white subretinal placoid lesions), (ii) retinal vasculitis (vascular sheathing, exudates, or hemorrhages), and (iii) confluent syphilitic retinochoroiditis (diffuse lesions with a “ground‐glass appearance” associated with yellow, small, preretinal precipitates). These diagnoses were validated by autofluorescence (AF; placoid hyperautofluorescence), optical coherence tomography (OCT; retinal pigment epithelial nodules, subretinal fluid, or irregularities), and fundus fluorescein/indocyanine green angiography (FFA/ICGA; extensive leakage and choroidal hypofluorescence, respectively). Second, optic nerve involvement (syphilitic optic neuritis) was clinically identified by optic disc edema, hyperemia, blurred margins, or peripapillary vascular tortuosity. These presentations were confirmed by extensive disc leakage on FFA and increased peripapillary retinal nerve fiber layer thickness on OCT.

To assess the infection status of syphilis and HIV, CD4^+^ T lymphocyte count and HIV viral load were collected, and serum, CSF (obtained via lumbar puncture), and AH (collected via anterior chamber paracentesis, 0.2 mL) tests for TPPA and RPR were performed, including both pre‐ and posttreatment results. Specifically, regarding AH retrieval, blood contamination was minimized via standardized anterior chamber paracentesis under microscopic visualization, strictly confining the entry site to the peripheral clear cornea anterior to the limbal vascular arcade. This ensured an avascular entry path and prevented iris injury, followed by the immediate exclusion of any samples exhibiting visible blood upon macroscopic inspection.

RPR reagents were obtained from the Shanghai Institute of Biological Products (Shanghai, China) and used strictly according to the manufacturer’s instructions. A standardized total AH volume of 100 μL was processed for each assay. Initially, a 50‐μL aliquot underwent a qualitative screen involving a 3‐min horizontal rotation and macroscopic assessment. Reactive samples utilized a separate 50‐μL aliquot for quantitative titration via twofold serial dilutions in 0.9% saline (undiluted to 1:32) under identical conditions. RPR titers ≥ 1:1 were considered reactive, whereas nonreactive samples were recorded as 1:0.5 and assigned a value of −1 for log_2_ transformation. RPR titers were further categorized as low (≤ 1:4), medium (≤ 1:16), and high (≥ 1:32) for subsequent analyses [14]. The logarithm of the minimum angle of resolution (logMAR) visual acuity values were derived from Snellen chart measurements. Visual acuities of counting fingers, hand movements, and light perception were assigned logMAR values of 1.9, 2.3, and 2.7, respectively [15].

Patients with confirmed diagnoses of ocular syphilis and AIDS were admitted, and all received antisyphilitic therapy in accordance with the Centers for Disease Control and Prevention guidelines for neurosyphilis. The treatment regimen consisted of intravenous aqueous crystalline penicillin G (3–4 million units every 4 h) for 10–14 days, followed by intramuscular benzathine penicillin G (2.4 million units weekly for three consecutive weeks) [16, 17]. In cases of severe vitreous haze with an inadequate response to antisyphilitic therapy, a local retrobulbar injection of methylprednisolone (20 mg) was administered as an adjunctive treatment [18]. RPR titers from serum, CSF, and AH, together with BCVA, were collected at baseline and at 6 months after treatment.

### 2.3. Statistical Analysis

Statistical analysis was performed using SPSS software (Version 25.0) and GraphPad Prism (Version 9.0.0). Descriptive statistics were used for demographic and clinical data. Continuous variables were expressed as mean ± standard deviation or median (interquartile range), and categorical variables as frequencies (%). The chi‐square test or Fisher’s exact test was used to compare categorical variables. Comparisons between two independent groups with non‐normally distributed data were performed using the Mann–Whitney *U* test. The correlations were evaluated using Spearman’s or Pearson’s correlation coefficient, as appropriate. Group differences in AH RPR titers across vitreous haze grades were analyzed using the Kruskal–Wallis test. When overall significance was detected, post hoc pairwise comparisons with appropriate corrections were performed to identify specific intergroup differences. Ordinal logistic regression analysis was performed to evaluate the association between pretreatment AH RPR titers and vitreous haze grade. The Wilcoxon matched‐pairs signed‐rank test was applied to compare paired data that did not follow a normal distribution. Statistical significance was defined as *p* < 0.05.

## 3. Results

A total of 42 eyes of 31 patients were included in this study. One eye in each patient was tapped for AH testing. For patients with bilateral involvement, the eye with more severe disease was selected for AH collection and subsequent analyses. Among the participants, 27 were male and 4 were female, with a median age of 33 (range, 26–43) years. All 31 patients had positive serum RPR results and underwent lumbar puncture, with CSF RPR positivity observed in 77.4% of cases. In addition, anterior chamber paracentesis was performed in one eye of each patient, yielding a 90.3% positivity rate for AH RPR. Among the cohort, 74.2% of patients had a detectable HIV viral load. The median CD4^+^ T lymphocyte count increased from 132 (range, 79–319) cells/μL before 6 months of antisyphilitic treatment to 217 (range, 84–405) cells/μL after treatment (Table 1).

**TABLE 1:** Characteristics of patients with ocular syphilis and AIDS.

Characteristics	*N* (%) or median (IQR)
Total patients	31 (42 eyes)
Bilateral involvement	11 (35.5)
Right	18 (42.9)
Left	24 (57.1)
Age (years)	33 (26–43)
Sex	
Male	27 (87.1)
Female	4 (12.9)
Anterior segment	
Anterior uveitis	7 (22.6)
Hypopyon	5 (16.1)
Intermediate segment	
Vitreous haze	
−	3 (9.7)
+	16 (51.6)
++	5 (16.1)
+++	7 (22.6)
Posterior segment	
Macular chorioretinal involvement	20 (64.5)
Optic nerve involvement	17 (54.8)
Reactive rate of RPR profiles	
Serum	31 (100)
CSF	24 (77.4)
AH	28 (90.3)
The HIV virus load	
Undetectable	8 (25.8)
Detectable	23 (74.2)
CD4^+^ T lymphocyte count before antisyphilitic treatment (cell/μL)	132 (79–319)
CD4^+^ T lymphocyte count after 6 months of treatment (cell/μL)	217 (84–405)
Perceived symptoms	
Yes	28 (90.3)
No	3 (9.7)

### 3.1. Pretreatment RPR Titers in Serum, CSF, and AH

As TPPA was uniformly positive in serum, CSF, and AH before treatment, it served primarily as a confirmatory test of infection. In all 31 patients, serum RPR testing was 100.0% reactive (31/31). By contrast, RPR reactivity in CSF was observed in 24 patients, corresponding to a reactivity rate of 77.4% (24/31), whereas in AH, 28 patients were reactive, yielding a reactivity rate of 90.3% (28/31) (Table 2). Notably, 3 cases were serum RPR reactive but AH RPR nonreactive (Figure 1(a)), and none exhibited hypopyon or anterior uveitis.

**TABLE 2:** Distribution of pretreatment RPR titers in serum, CSF, and AH by titer level category.

	**NR (< 1:1)**	**R: low (≤ 1:4)**			**R: medium (≤ 1:16)**		**R: high (≥ 1:32)**			

Biological fluid	< 1:1	1:1	1:2	1:4	1:8	1:16	1:32	1:64	1:128	1:256
Serum	0 (0.0%)	0 (0.0%)	0 (0.0%)	0 (0.0%)	3 (9.7%)	1 (3.2%)	7 (22.6%)	11 (35.5%)	4 (12.9%)	5 (16.1%)
CSF	7 (22.6%)	0 (0.0%)	5 (16.1%)	6 (19.4%)	10 (32.3%)	1 (3.2%)	1 (3.2%)	1 (3.2%)	0 (0.0%)	0 (0.0%)
AH	3 (9.7%)	0 (0.0%)	4 (12.9%)	9 (29.0%)	6 (19.4%)	4 (12.90%)	3 (9.7%)	2 (6.4%)	0 (0.0%)	0 (0.0%)

*Note:* NR: nonreactive; R: reactive.

FIGURE 1Pretreatment RPR profiles in serum, CSF, and AH. (a) Venn diagram illustrating the overlap of reactive and nonreactive RPR testing results among serum, CSF, and AH samples. (b) Correlation between serum and CSF RPR titers. (c) Correlation between serum and AH RPR titers. (d) Correlation between AH and CSF RPR titers.

**Figure:**
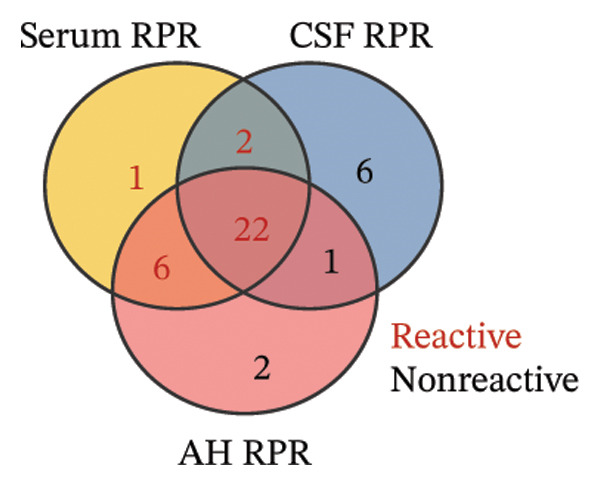
(a)

**Figure:**
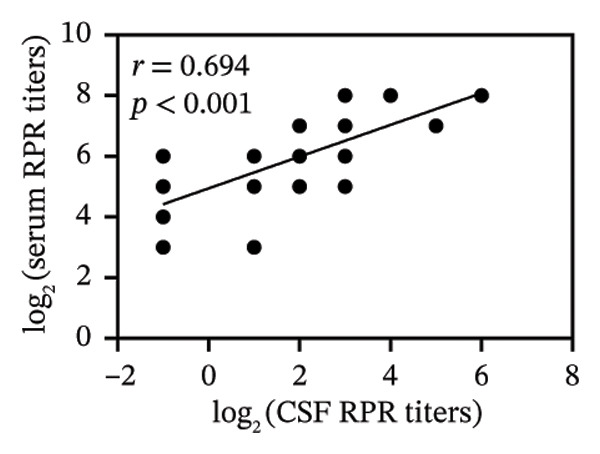
(b)

**Figure:**
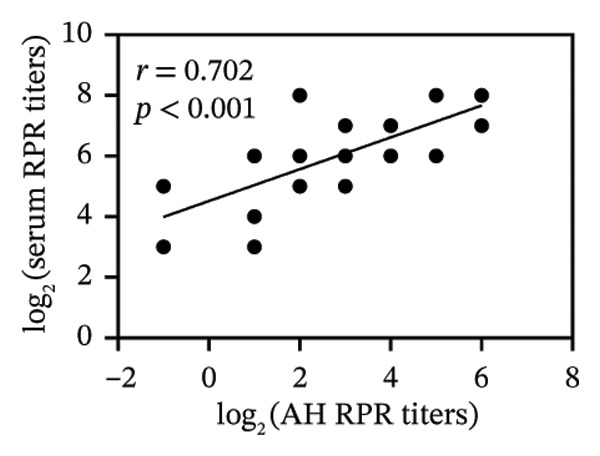
(c)

**Figure:**
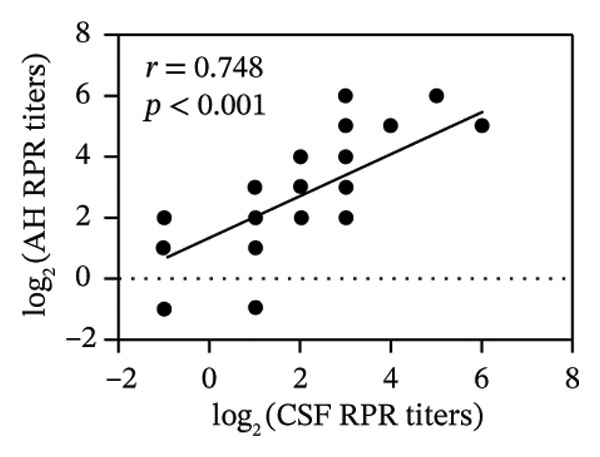
(d)

We next analyzed the distribution of titers, and pretreatment serum RPR showed high titers (≥ 1:32) in 87.1% (27/31) and medium titers (≤ 1:16) in 12.9% (4/31). By contrast, CSF showed attenuation with 22.6% (7/31) nonreactive and only 6.5% (2/31) high titers, while AH demonstrated a similar but slightly more reactive profile, with 9.7% (3/31) nonreactive and 16.1% (5/31) high titers (Table 2). The median log_2_‐transformed pretreatment RPR titer in serum (6; range, 5–7) was significantly higher than that in CSF (2; range, 1–3; *p* < 0.001) and AH (2; range, 2–4; *p* < 0.001). These findings emphasize the higher sensitivity of serum testing compared with CSF and AH. Regarding RPR titer in serum, CSF, and AH, Spearman correlation analysis revealed significant positive associations between serum and CSF RPR titers (*r* = 0.69, *p* < 0.001), serum and AH RPR titers (*r* = 0.70, *p* < 0.001), and AH and CSF RPR titers (*r* = 0.75, *p* < 0.001) (Figures 1(b), 1(c), and 1(d)).

### 3.2. Pretreatment RPR Titer Profiles in Relation to Severity of Anterior Segment Involvement

Among the 31 eyes, 7 presented with anterior uveitis and 5 with anterior chamber hypopyon. In eyes with anterior uveitis, pretreatment log_2_‐transformed RPR titers were significantly elevated across all three biological fluids, with a serum median of 7 (range, 6.5–8), CSF median of 3 (range, 3–3.5), and AH median of 4 (range, 3.5–5). By comparison, eyes without anterior uveitis showed lower titers—serum median 6 (range, 5–6; *p* = 0.004), CSF median 2 (range, −1–2.25; *p* < 0.001), and AH median 2 (range, 1–3; *p* < 0.001). Similarly, in eyes with anterior chamber hypopyon, serum, CSF, and AH RPR titers were also markedly higher, with medians of 7 (range, 7–8), 4 (range, 3–5), and 5 (range, 4–5), respectively. In contrast, eyes without hypopyon demonstrated lower titers—serum median 6 (range, 5–6; *p* = 0.002), CSF median 1.5 (range, −0.5–3; *p* < 0.001), and AH median 2 (range, 1.25–3; *p* = 0.003).

### 3.3. Pretreatment RPR Titer Profiles in Relation to the Severity of Intermediate Segment Involvement

Among 31 patients, those without vitreous haze showed high‐titer serum RPR, whereas AH RPR was nonreactive in 33.3% (1/3) and medium in the remainder, with no high titers observed (Figure 2(a) and Table S1). Correlation analysis showed that serum RPR reactivity levels were not significantly associated with vitreous haze grades (*r* = 0.21, *p* = 0.256) (Figure 2(b)). In contrast, pretreatment CSF and AH RPR reactivity levels were positively correlated, with AH demonstrating a stronger association (*r* = 0.54, *p* = 0.002) than CSF (*r* = 0.41, *p* = 0.023) (Figures 2(c) and 2(d)). Moreover, the Kruskal–Wallis test demonstrated significant differences in AH RPR titers across vitreous haze grades (*p* = 0.005), with post hoc analysis confirming that the most pronounced difference was between haze Grade 1 and Grade 3 (*p* = 0.008). Notably, the median AH RPR titer increased from 1:4 in eyes with Grade 1 haze to 1:16 in eyes with Grade 3 haze, indicating that higher AH RPR titers were associated with more severe vitreous haze grades. Ordinal logistic regression analysis showed that pretreatment AH RPR titer was associated with vitreous haze grade (OR = 4.10, 95% CI: 1.62–10.38, *p* = 0.003).

FIGURE 2Pretreatment serum and AH RPR titers across vitreous haze grades. (a) Box plots showing the RPR titers across different grades of vitreous haze. Serum RPR titers are shown in yellow, and AH RPR titers are shown in red. (b) Correlation between serum RPR reactivity levels and vitreous haze grades (NR = 0, R: low = 1, R: medium = 2, and R: high = 3). (c) Correlation between CSF RPR reactivity levels and vitreous haze grades. (d) Correlation between AH RPR reactivity levels and vitreous 2haze grades.

**Figure:**
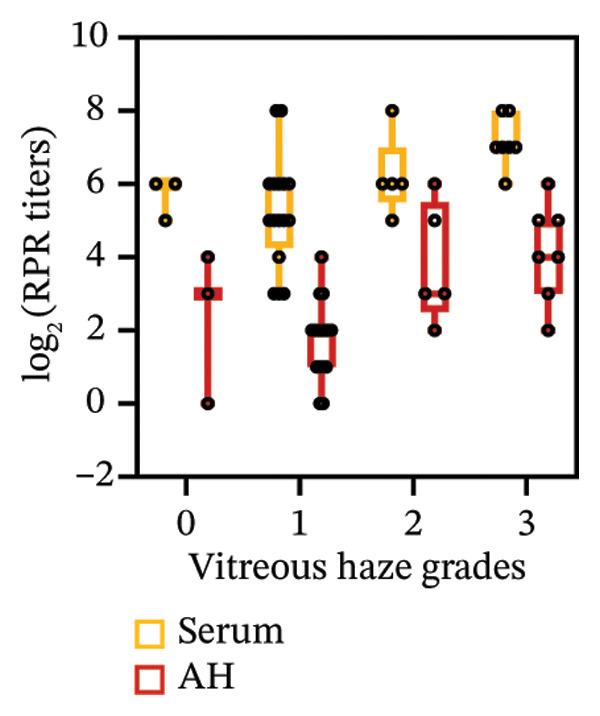
(a)

**Figure:**
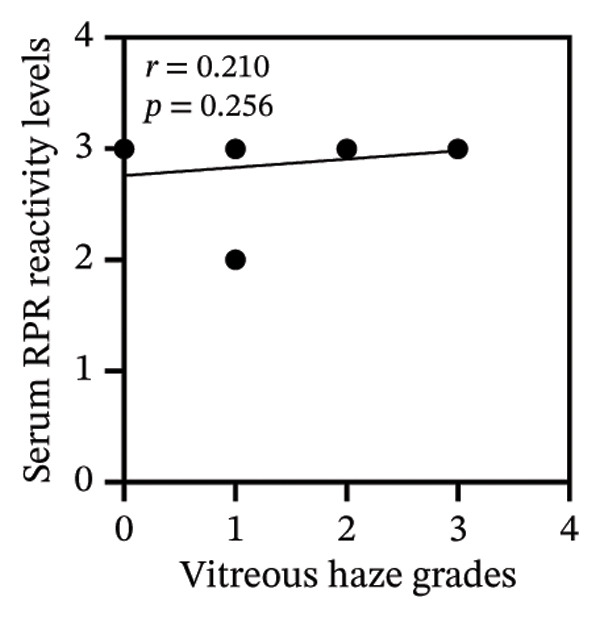
(b)

**Figure:**
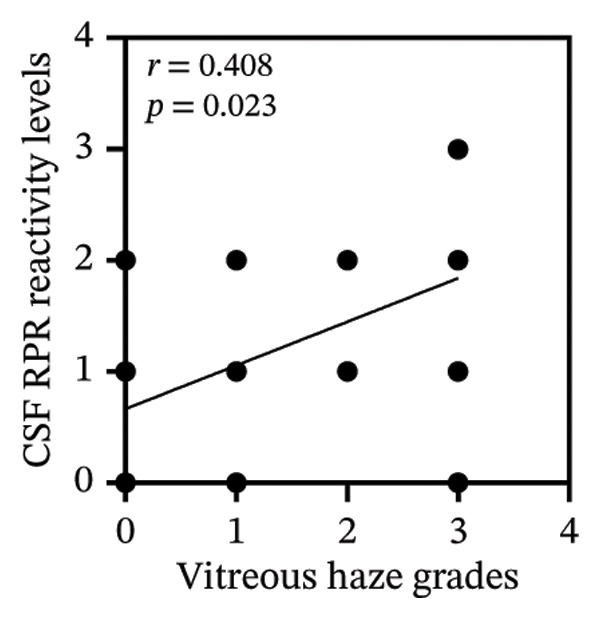
(c)

**Figure:**
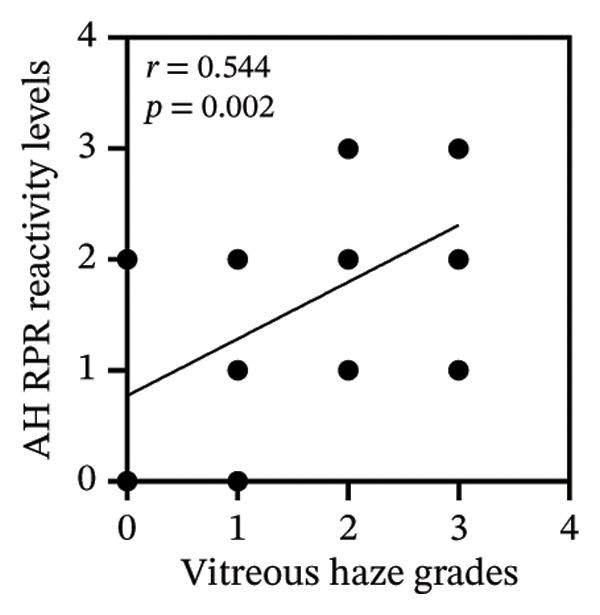
(d)

### 3.4. Pretreatment RPR Titer Profiles in Relation to the Severity of Posterior Segment Involvement

Among 31 eyes, 20 showed macular chorioretinal involvement. Pretreatment RPR reactivity was 100.0% in serum, 90.0% in CSF, and 95.0% in AH (Table S2). ROC analysis evaluated the predictive value of RPR titers for macular chorioretinal involvement. Individually, pretreatment serum, CSF, and AH RPR titers yielded AUCs of 0.84 (*p* = 0.002), 0.85 (*p* = 0.001), and 0.76 (*p* = 0.021), with the optimal log_2_ cutoff values of 6.5, 1.5, and 1.5, respectively (Figures 3(a), 3(b), and 3(c)). Logistic regression combining serum and CSF, as well as all three markers, improved discrimination (AUC 0.88, *p* < 0.001; AUC 0.88, *p* = 0.001, respectively), with probability thresholds of 0.49 and 0.82 (Figures 3(d) and 3(e)).

FIGURE 3Receiver operating characteristic (ROC) curve of serum RPR titers, CSF RPR titers, AH RPR titers, and their combination for predicting posterior segment involvement. (a–e) ROC curves of pretreatment RPR titers in serum (a), CSF (b), AH (c), their combination in serum and CSF (d), and the combination of serum, CSF, and AH (e) for predicting macular chorioretinal involvement. (f–j) ROC curves of pretreatment RPR titers in serum (f), CSF (g), AH (h), their combination in serum and CSF (i), and the combination of serum, CSF, and AH (j) for predicting optic nerve involvement.

**Figure:**
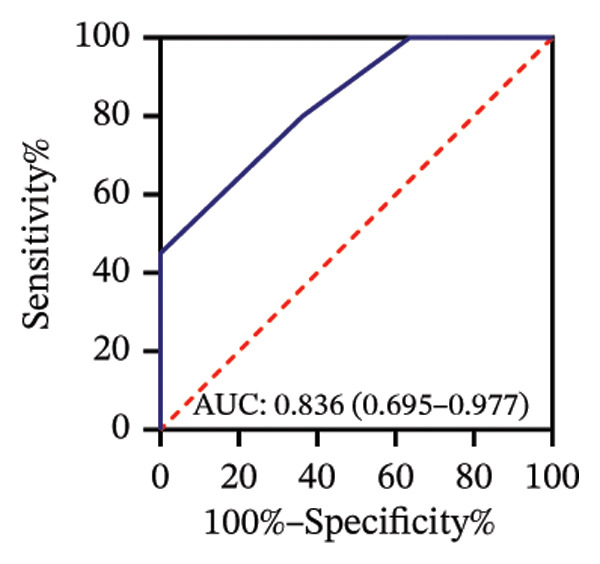
(a)

**Figure:**
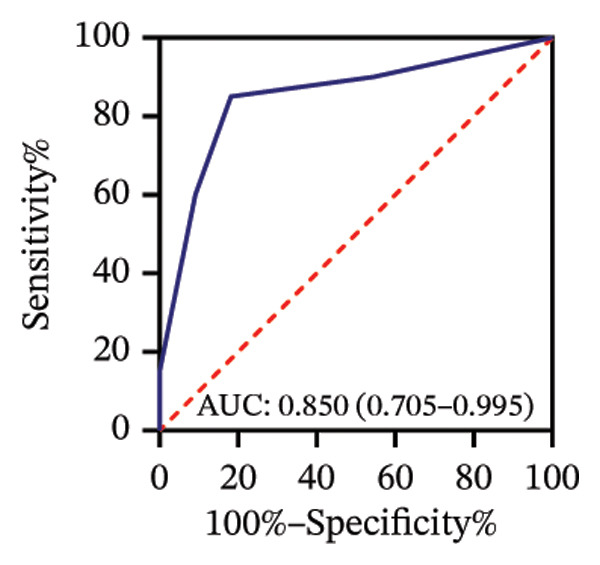
(b)

**Figure:**
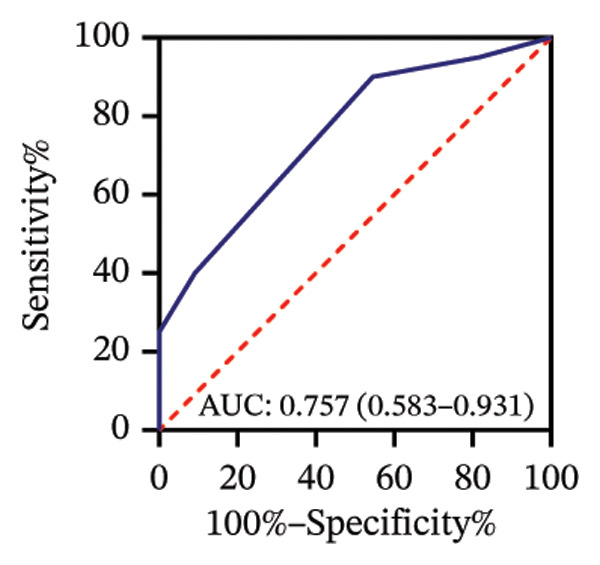
(c)

**Figure:**
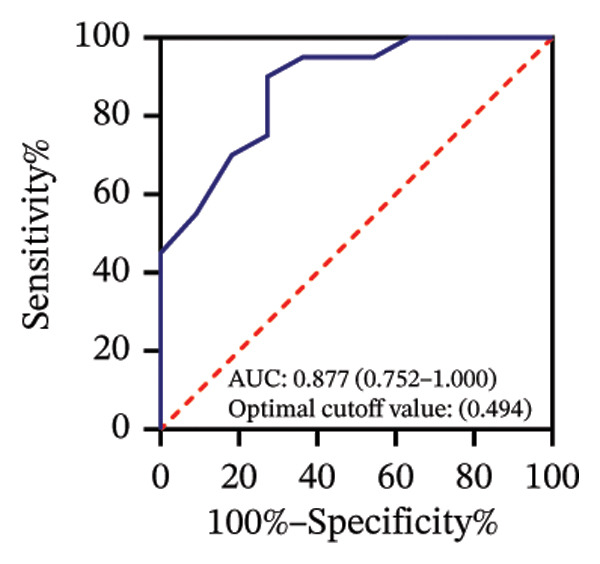
(d)

**Figure:**
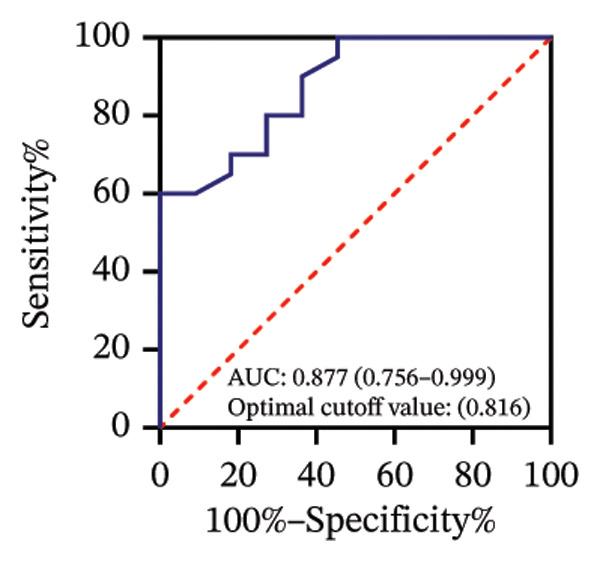
(e)

**Figure:**
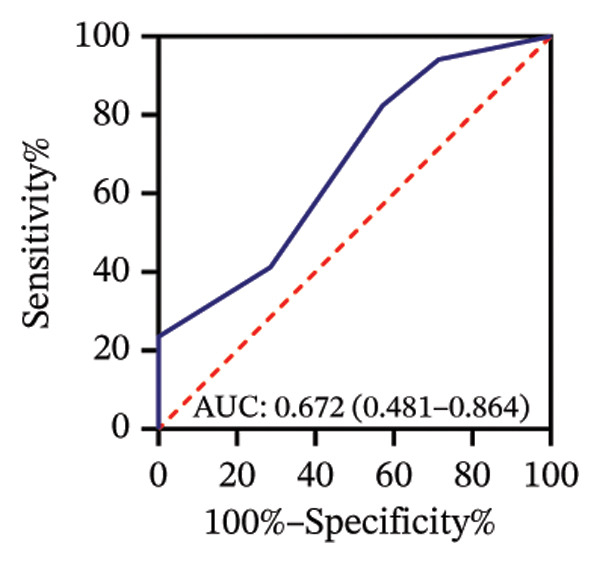
(f)

**Figure:**
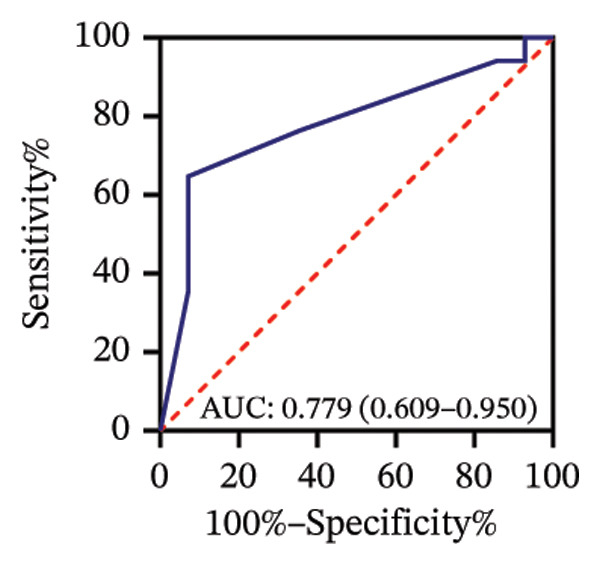
(g)

**Figure:**
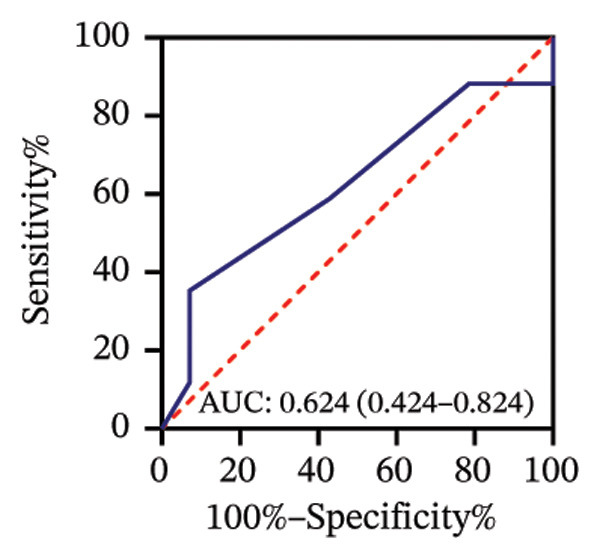
(h)

**Figure:**
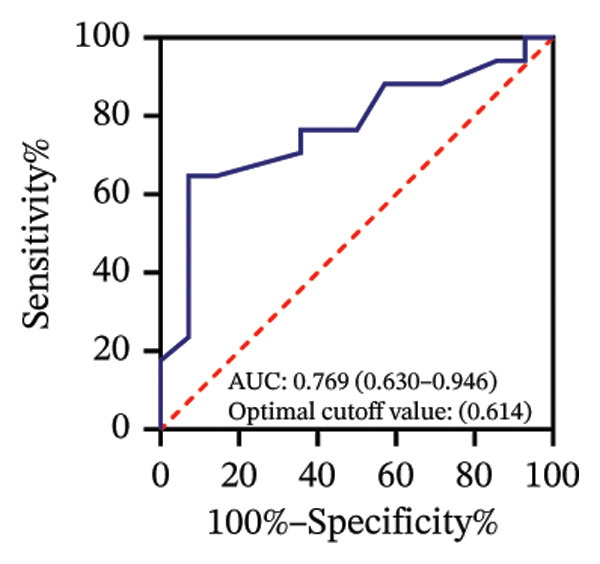
(i)

**Figure:**
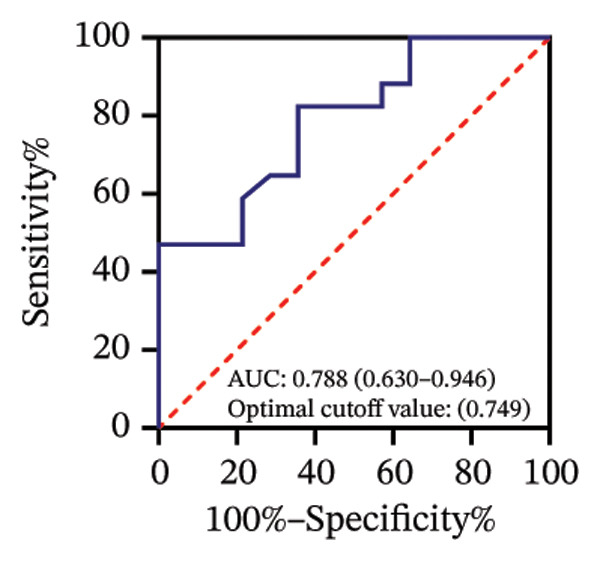
(j)

Among 31 eyes, 17 showed optic nerve involvement. Before treatment, the RPR reactivity rates in serum, CSF, and AH were 100.0%, 64.7%, and 88.2%, respectively (Table S2). ROC analysis evaluated the predictive value of RPR titers for optic nerve involvement. Individually, pretreatment serum, CSF, and AH RPR titers yielded AUCs of 0.67 (*p* = 0.104), 0.78 (*p* = 0.008), and 0.62 (*p* = 0.242), with the optimal log_2_ cutoff values of 6.5, 1.5, and 1.5, respectively (Figures 3(f), 3(g), and 3(h)). Logistic regression combining serum and CSF RPR titers improved discrimination (AUC = 0.77, *p* < 0.001), and inclusion of all three markers further enhanced predictive accuracy (AUC = 0.79, *p* < 0.001), with probability thresholds of 0.61 and 0.75, respectively (Figures 3(i) and 3(j)).

### 3.5. Association of BCVA With AH RPR

The median pretreatment BCVA (logMAR) was 1.0 (range, 0.6–1.7), which improved to a median of 0.7 (range, 0.3–1.0) after treatment (*p* = 0.024).

Pretreatment BCVA showed significant correlations with pretreatment RPR titers in serum, CSF, and AH (*r* = 0.71, *p* < 0.001; *r* = 0.53, *p* = 0.002; and *r* = 0.47, *p* = 0.007, respectively) (Figures 4(a), 4(b), and 4(c)). Moreover, posttreatment BCVA was significantly correlated with pretreatment RPR titers in serum, CSF, and AH (*r* = 0.67, *p* < 0.001; *r* = 0.54, *p* = 0.002; and *r* = 0.45, *p* = 0.012, respectively) (Figures 4(d), 4(e), and 4(f)). In addition, posttreatment BCVA was strongly correlated with pretreatment BCVA (*r* = 0.82, *p* < 0.001) (Figure 4(g)). These findings suggest that RPR levels in biological fluids may reflect visual outcomes.

FIGURE 4Association of visual acuity outcomes with pretreatment RPR titers in three biological fluids. (a–c) Correlation between pretreatment BCVA and pretreatment RPR titers in three biological fluids, including serum (a), CSF (b), and AH (c). (d–f) Correlations between posttreatment BCVA and pretreatment RPR titers in three biological fluids, including serum (d), CSF (e), and AH (f). (g) Correlation between posttreatment BCVA and pretreatment BCVA.

**Figure:**
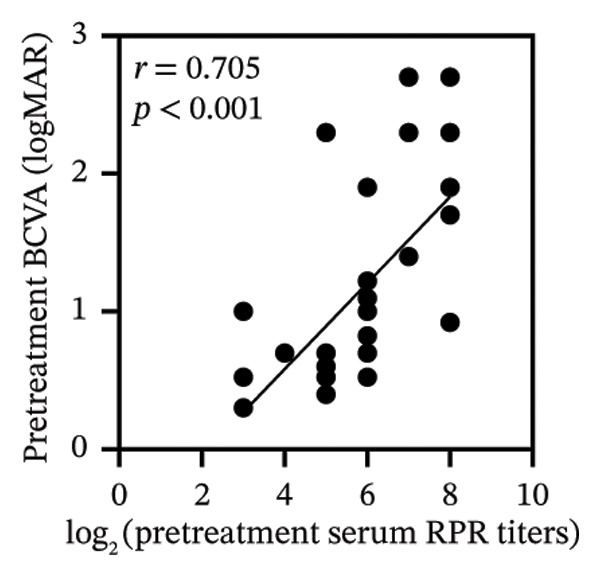
(a)

**Figure:**
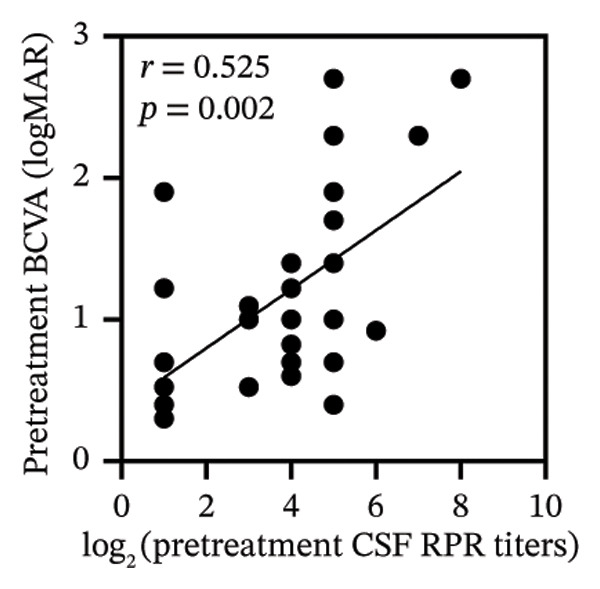
(b)

**Figure:**
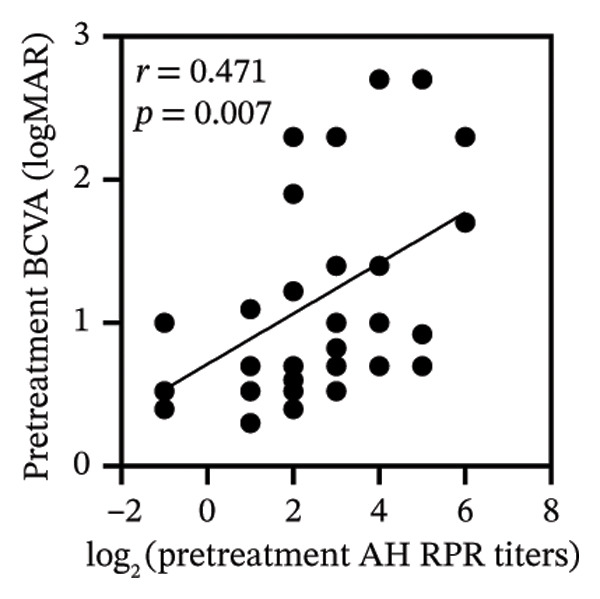
(c)

**Figure:**
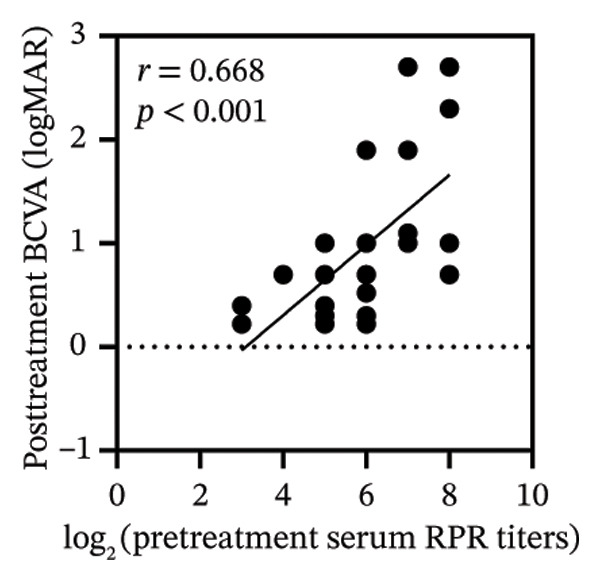
(d)

**Figure:**
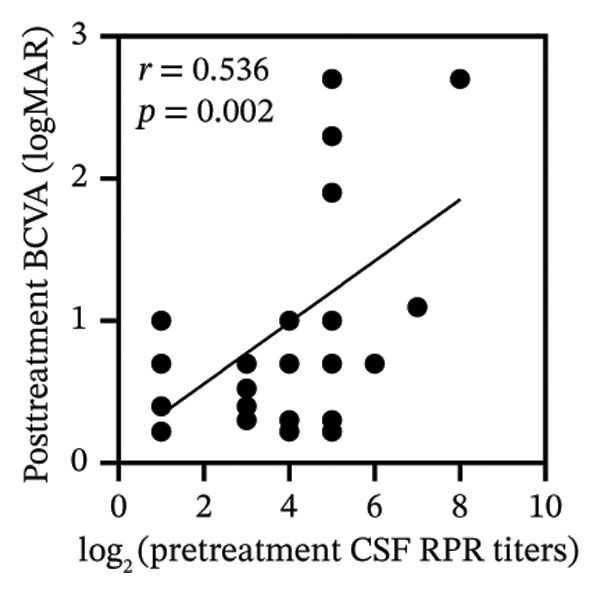
(e)

**Figure:**
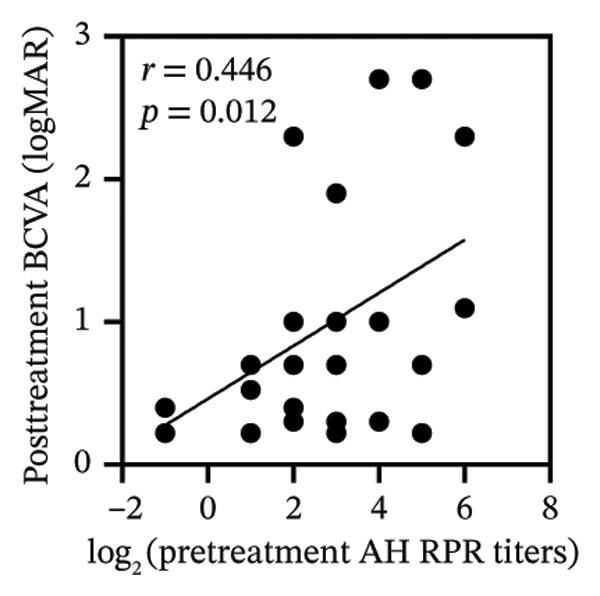
(f)

**Figure:**
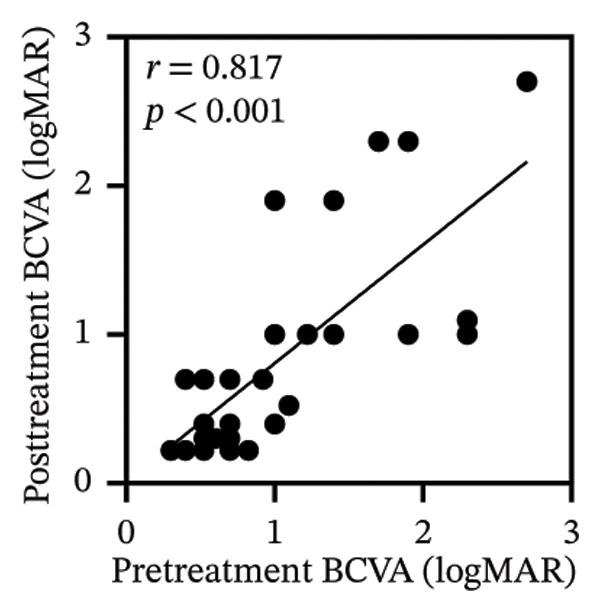
(g)

Following treatment, RPR nonreactivity was achieved in 71.0% (22/31) of serum samples, 90.3% (28/31) of CSF samples, and 87.1% (27/31) of AH samples. Among the 29.0% (9/31) of patients who remained serum RPR reactive, 25.8% (8/31) had low‐to‐moderate titers and 3.2% (1/31) had high titers, whereas the 9.7% (3/31) of CSF cases and 12.9% (4/31) of AH cases that failed to reach nonreactivity were all confined to the low‐titer group. In parallel, the mean log_2_‐transformed RPR titers decreased significantly after treatment across all three biological fluids: from 5.87 to 0.06 in serum (*p* < 0.001), from 1.77 to −0.84 in CSF (*p* < 0.001), and from 2.58 to −0.71 in AH (*p* < 0.001). Visual outcomes also improved, with 54.8% (17/31) of eyes showing a clinically significant gain in BCVA, defined as a decrease of more than 0.2 logMAR [15]. Among these patients with improved visual acuity, RPR nonreactivity was achieved in 82.4% (14/17) of serum samples, 88.2% (15/17) of CSF samples, and 76.5% (13/17) of AH samples.

## 4. Discussion

In this study, we evaluated RPR titers in serum, CSF, and AH in patients with ocular syphilis and AIDS to investigate their clinical utility and associations with ocular manifestations and visual outcomes. We found that serum RPR titers were not correlated with vitreous haze grades, whereas AH RPR titers demonstrated a significant positive correlation (*r* = 0.54, *p* = 0.002) that was stronger than that of CSF (*r* = 0.41, *p* = 0.023). Moreover, higher reactivity levels of AH RPR were associated with an increased risk of greater vitreous haze grade (OR = 4.10, 95% CI: 1.62–10.38, *p* = 0.003). Although the serum RPR test demonstrated higher sensitivity (100.0%) than CSF (77.4%) and AH (90.3%) RPR tests (Table 1), its clinical utility in evaluating ocular disease activity is limited, as it may not accurately reflect intraocular pathological burden such as vitreous haze. This finding suggests that reliance on serum RPR alone may lead to misinterpretation of disease activity, underscoring the value of AH testing for accurate assessment.

Previous studies have shown that a reactive CSF RPR indicates *Treponema pallidum* invasion of the central nervous system and may reflect the involvement of the visual pathways in ocular syphilis [19–21]. AH and CSF RPR titers were strongly correlated (*r* = 0.75, *p* < 0.001), which is consistent with previous studies. We found that, in the ROC analysis, the optimal probability threshold for predicting macular chorioretinal involvement was 0.82 when combining serum, CSF, and AH RPR, corresponding to a specificity of 100%, whereas the optimal threshold was 0.49 for the combination of serum and CSF RPR, with a specificity of 72.7%. These findings highlight that combining AH RPR better captures the local ocular features of the disease. A similar pattern was observed in the ROC analysis for optic nerve involvement. However, although the AUC of the combined analysis of all three markers was higher than that of any single marker, it showed a little or no improvement compared with the combination of serum and CSF RPR. This may suggest that AH RPR provides limited additional information regarding posterior segment involvement.

Our analysis demonstrated that both pre‐ and posttreatment BCVA were significantly correlated with RPR titers in serum, CSF, and AH, indicating that worse visual acuity is associated with higher RPR titers. Elevated titers likely reflect greater pathogen activity and a stronger potential for tissue damage, which may contribute to impaired vision. Furthermore, posttreatment outcomes were strongly correlated with pretreatment BCVA, consistent with previous reports, suggesting that patients with better initial vision tend to achieve better visual recovery. These findings collectively emphasize that early and targeted intervention in ocular syphilis is critical for optimizing visual function.

In clinical practice, AH RPR serves as a pivotal localized biomarker that signifies active intraocular syphilitic infection or a critically heightened risk of treponemal invasion. This indicator effectively addresses the diagnostic ambiguity where a positive serum RPR reflects a broad systemic background, a limitation particularly critical in patients with AIDS who often present with complex, coexisting opportunistic infections. Furthermore, AH RPR titers offer a reflection of local pathological burden, as exemplified by their correlation with vitreous haze grades and enhanced predictive performance for posterior segment involvement. Ultimately, AH RPR optimizes the diagnostic strategy by shifting the focus from general systemic status to precise *in situ* ocular localization, facilitating individualized precision management for patients with AIDS and concurrent ocular syphilis.

## 5. Conclusion

This study establishes AH RPR as a pivotal localized biomarker for the diagnosis of ocular syphilis, particularly among patients with AIDS. The clinical utility of this marker is exemplified by its significant correlation with vitreous haze grades and its capacity to enhance the predictive performance of diagnostic models for posterior segment involvement. While AH RPR may originate from either *in situ* antibody synthesis or passive diffusion across a disrupted blood–ocular barrier, both mechanisms signify an active intraocular syphilitic infection or a critically heightened risk of treponemal invasion. Therefore, this localized indicator maintains substantial clinical significance by shifting the diagnostic focus from a general systemic background to precise *in situ* ocular involvement. The incorporation of AH RPR into clinical evaluation facilitates a more accurate diagnosis of ocular syphilis, guides tailored therapeutic strategies, and ultimately improves functional visual outcomes.

## Author Contributions

The study was designed and conducted by Wenjun Kong, Yong Tao, and Zhuyun Qian. Data collection, management, analysis, and interpretation were performed by Wenjun Kong, Zhuyun Qian, and Yingjie Wang. The manuscript was prepared by Yingjie Wang. Wenjun Kong, Yong Tao, and Zhuyun Qian revised the manuscript.

## Funding

This study was supported by the “Youth” Project of Beijing Hospital Management Center (QML20211703); National Natural Science Foundation of China (Nos. 82525020 and 82471081); Excellent Young Talent Innovation Project and grants (CX23YQ03, KCA2302, and CX23YQA02) from the Chinese Institutes for Medical Research, Beijing; and Beijing Nova Program (No. 202304844450) from the Chinese Institutes for Medical Research, Beijing.

## Ethics Statement

This study followed the Declaration of Helsinki and was approved by Beijing You’an Hospital.

## Conflicts of Interest

The authors declare no conflicts of interest.

## Supporting Information

Table S1: Distribution of pretreatment AH RPR titers across vitreous haze grades. Table S2: RPR titer profiles in relation to severity of posterior segment involvement.

## Supporting information


**Supporting Information** Additional supporting information can be found online in the Supporting Information section.

## Data Availability

All data examined in the current study can be provided from the corresponding author upon reasonable request.
