# Supplementary material for: Rapid Plasma Reagin Tests of Serum, Cerebrospinal Fluid, and Aqueous Humor of Patients With Ocular Syphilis and AIDS
Source: J Ophthalmol. 2026 Apr 22;2026:5291594. doi: 10.1155/joph/5291594 (PMC13101952; doi:10.1155/joph/5291594)
Supplement: Supplementary file 1 — Supporting Information Additional supporting information can be found online in the Supporting Information section. [file JOPH-2026-5291594-s001.docx]

**Supporting Information**

Table. S1. Distribution of pre-treatment AH RPR titers across vitreous haze grades.

|  | Vitreous haze grades | | | |
| --- | --- | --- | --- | --- |
| AH | 0 | 1 | 2 | 3 |
| NR | 1 (33.3%) | 0 (0.0%) | 0 (0.0%) | 0 (0.0%) |
| R: low | 0 (0.0%) | 2 (12.5%) | 1 (20.0%) | 1 (14.2%) |
| R: medium | 2 (66.7%) | 11 (68.8%) | 2 (40.0%) | 3 (42.9%) |
| R: high | 0 (0.0%) | 3 (18.7%) | 2 (40.0%) | 3 (42.9%) |
| Summary | 3 (100.0%) | 16 (100.0%) | 5 (100.0%) | 7(100.0%) |

NR: nonreactive; R: reactive.

Table. S2. RPR titer profiles in relation to severity of posterior segment involvement.

|  |  | Macular chorioretinal involvement (Yes/No) | | Optic nerve involvement  (Yes/No) | |
| --- | --- | --- | --- | --- | --- |
|  |  | No | Yes | No | Yes |
| Serum | R: Medium | 4 (36.4%) | 0 (0.0%) | 0 (0.0%) | 4 (23.5%) |
|  | R: High | 7 (63.6%) | 20 (100.0%) | 14 (100.0%) | 13 (76.5%) |
| CSF | NR | 5 (45.5%) | 2 (10.0%) | 1 (7.1%) | 6 (35.3%) |
|  | R: Low | 5 (45.5%) | 6 (30.0%) | 4 (28.6%) | 7 (41.2%) |
|  | R: Medium | 1 (9.1%) | 10 (50.0%) | 8 (57.1%) | 3 (17.6%) |
|  | R: High | 0 (0.0%) | 2 (10.0%) | 1 (7.1%) | 1 (5.9%) |
| AH | NR | 2 (18.2%) | 1 (5.0%) | 1 (7.1%) | 2 (11.8%) |
|  | R: Low | 6 (54.5%) | 7 (35.0%) | 5 (35.7%) | 8 (47.1%) |
|  | R: Medium | 3 (27.3%) | 7 (35.0%) | 5 (35.7%) | 5 (29.4%) |
|  | R: High | 0 (0.0%) | 5 (25.0%) | 3 (21.5%) | 2 (11.7%) |

NR: nonreactive; R: reactive.
